# Salt‐tolerant *Staphylococcus* bacteria induce structural and nutritional alterations of salted duck egg white

**DOI:** 10.1002/fsn3.1255

**Published:** 2019-11-07

**Authors:** Gongnian Xiao, Yuanzhe Chen, Ruosi Fang, Chaogeng Xiao, Haina Yuan, Bingquan Chu, Xuangan Liu, Jinyan Gong

**Affiliations:** ^1^ Zhejiang Provincial Key Lab for Biological and Chemical Processing Technologies of Farm Products School of Biological and Chemical Engineering Zhejiang University of Science and Technology Hangzhou China; ^2^ Institute of Food Science Zhejiang Academy of Agricultural Sciences Hangzhou China

**Keywords:** 16S rRNA, fermentation, salted duck egg white, *Staphylococcus*, γ‐aminobutyric acid

## Abstract

Salted duck egg white, a major by‐product of salted egg yolk production, is rich in nutrients. However, its high salinity limits its application in the food industry. In the present study, three haloduric bacterium strains (C1, C2, and C3) were isolated from Jinhua ham, and strain C1 exhibited higher ratio of the transparent circle diameter to the colony diameter (HC) and gelatin liquefaction. Strain C1 was further identified as a member of the genus *Staphylococcus* through gene sequencing and EzTaxon‐e analyses. Salted duck egg white was fermented by strain C1, and the thermal stability, microstructure, amino acid composition, and γ‐aminobutyric acid of the egg white were compared with egg white without fermentation. The fermented salted duck egg white had a significantly low salinity. Meanwhile, it increased its thermal stability compared with the control through losing an endotherm at around 85°C and forming a new endotherm peak starting at 91.8°C. Additionally, free amino acids and γ‐aminobutyric acid were found only in the fermented salted duck egg white. These indicated that fermentation with salt‐resistant strains could alter the structure of salted duck egg white and improve its nutritional quality.

## Graphical abstract

A *Staphylococcus* strain was isolated from Jinhua ham, which enhanced thermal stability and altered microstructure of salted duck egg white by fermentation. Free amino acids and γ‐aminobutyric acid were found in fermented salted duck egg white.

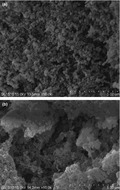

## 1 INTRODUCTION

Salted duck eggs are one of the most famous Chinese preserved food products. Normally, salted duck eggs are made through soaking eggs in brine solution to alter the structure and flavor of duck eggs (Kaewmanee, Benjakul, & Visessanguan, 2009a; Lin, 2014). China has produced tens of thousands of salted duck eggs annually, and it is considered the largest salted duck eggs producer in the world (Adzitey & Adzitey, 2011). Salted duck egg yolk possesses orange color and unique aroma, and it is normally used in Chinese mooncakes (Yang et al., 2015). Salted duck egg white (SDEW) is a major by‐product generated from the salted duck egg yolk production and its high level of vitamins, minerals, and amino acids make it a good food source (Kaewmanee, Benjakul, & Visessanguan, 2009b). However, a high salinity has limited its application in the food industry and a desalination process of salted duck egg white is conducted before incorporating salted duck egg white as an ingredient in food products (Wang, Zhang, Adhikari, Mujumdar, & Zhou, 2013). Many strategies on the desalination of salted duck egg white have been studied recently. For example, it has been reported that an ultrafiltration technique was applied to significantly desalinate the salinity and preserve the protein content of salted duck egg white (Hou, Liu, Shi, Ma, & He, 2017; Zhou, Zhang, Fang, & Liu, 2015). Moreover, salted duck egg white after an electrodialysis desalination process has been reported to reduce 95% salt percentage and recover about 90% of protein content (Huang, Tsai, & Pan, 1999). What is more, a membrane desalination technology has been applied to salted duck egg white to reduce its salt level (Zhao et al., 2014). However, the sticky texture of salted duck egg white could result in clogs of membranes during the desalination process. Meanwhile, the nutrients left on the membrane could cause microorganism spoilage (Zhao et al., 2014). Therefore, such drawbacks limit the application of membrane desalination on salted duck egg whites. Enzymatic hydrolysis has further been applied to salted duck egg white before the membrane desalination process to prevent the membrane clogs through decreasing the viscosity of salted duck egg white (Hou et al., 2017). However, high salinity of salted duck egg white could significantly reduce the activity of proteases and the hydrolysates after hydrolysis could exert a negative effect on desalination (Ganesan, Kaewmanee, Benjakul, & Baharin, 2014). Fermentation has been widely used in the food industry, and it has gained attention on desalination of salted duck egg white. Compared with other desalination methods, fermentation could provide multiple benefits. For example, microorganisms during fermentation process could release various enzymes that could possess microbial protease production and enzymolysis. These could significantly reduce the viscosity of salted duck egg white through degrading the macromolecular egg white proteins to improve filtration process (Ganesan et al., 2014). Meanwhile, fermentation process is a simple process with a low cost, and it could easily recycle salted duck egg whites after process. The egg white after the fermentation could result in the release of numerous bioactive proteins and peptides, and these bioactive compounds could be incorporated in foods to significantly enhance the value of nutrients in foods. Due to its high salinity, a bacterium strain with high salinity tolerance is required to accomplish fermentation process. In the present study, a novel *Staphylococcus* was found and isolated from Jinhua ham that possessed high salt‐tolerant feature. We inoculated this isolate into salted duck egg white to initiate fermentation, and the physical and chemical properties of salted duck egg white were analyzed. The findings from this study could provide a useful reference on desalinization of salted duck egg white and improvement of nutritional value of desalinized salted duck egg white.

## 2 MATERIALS AND METHODS

### 2.1 Materials and chemicals

Jinhua ham was provided from Jinzi Ham Co. Ltd in 2017. The *Staphylococcus* culture media used in this study included International Streptomyces Project (ISP) medium 1, ISP medium 2, ISP medium 3, and ISP medium 4. These culture media were purchased from Sinopharm Chemical Reagent Co. Ltd. ISP medium 1 contained 3 g of beef extract, 10 g of peptone, 50 g of sodium chloride in 1,000 ml water with a 7.4–7.6 pH value, whereas ISP medium 2 was formularized with 1 g beef extract, 10 g peptone, 10 g d‐mannitol, 100 g sodium chloride, 2.5 ml 1% phenol red, and 15–20 g agar in 1,000 ml water with a 7.2–7.6 pH condition. ISP medium 3 consisted of 16 g casein, 30 g cane sugar, 1 g K_2_HPO_4_, 75 g sodium chloride, 0.5 g MgSO_4_, 2 g KNO_3_, 0.01 g FeSO_4_, 15–20 g agar, and 1,000 ml water (pH 7.0). ISP medium 4 was comprised of 3 g beef extract, 10 g peptone, 75 g sodium chloride, and 200 g gelatin in 1,000 ml water (pH 7.2–7.4). All external amino acids were purchased from FUJIFILM Wako Pure Chemical Corporation with a purity above 98%. The external standard γ‐aminobutyric acid, with a 98% purity, was purchased from J&K Chemical Technology Co. Ltd.

### 2.2 Strain culture and isolation

The Jinhua ham (10.0 g) was inoculated into 50 ml of ISP medium 1 under an aseptic condition. The inoculated medium was incubated at 37°C for 24 hr. The bacteria suspension (1 ml, from ISP medium 1) was diluted and then passed to the ISP medium 2 using streak plates, followed by the incubation at 37°C for 48 hr. Three haloduric bacteria strains C1, C2, and C3 were isolated due to their growth and colony appearance (yellowish color colony) in the ISP medium 2. Afterward, these strains were passed into ISP medium 3 to test their protease‐producing activity, whereas these strains inoculated in ISP medium 4 were used to investigate the highest HC and gelatin liquefaction. Haloduric bacteria C1 were screened to be the best strain regarding its protease‐producing activity, highest HC degree, and gelatin liquefaction. Therefore, the consumption of carbon source and nitrogen source of the strain C1 was analyzed by supplementing the medium with various tested sources at a final concentration 1% and 0.1% (w/v), respectively. The carbon and nitrogen source media were supplemented with 10% (w/v) sodium chloride.

### 2.3 Gene sequence

The strain C1 after strain culture was immediately delivered to Sangon Biotech Co. Ltd for strain determination. In brief, the extraction of the DNA in the strain C1 was extracted using the SK8255 (bacterium), SK8259 (fungus), and SK8257 (yeast) kit and the extraction procedure followed the kit instruction. PCR amplification was carried out using bacterial 16S general primer (27F: AGTTTGATCMTGGCTCAG, 1492R: GGTTACCTTGTTACGACTT), fungal ITS general primer (ITS1: TCCGTAGGTGAACCTGCGG, ITS4: TCCTCCGCTTATTGATATGC), 18 S general primer (NS1: GTAGTCATATGCTTGTCTC, NS6: GCATCACAGACCTGTTATTGCCTC), and yeast 26S general primer (NL4: GCATATCAATAAGCGGAGGAAAAG, NL4: GGTCCGTGTTTCAAGACGG), respectively. The amplification system consisted of 0.5 µl extracted DNA, 2.5 µl buffer containing 2.5 mM Mg^2+^, 0.2 µl Taq polymerase, 1.0 µl of 10 mM dNTP, 0.5 µl of 10 µM forward primer, 0.5 µl of 10 µM reverse primer, and 25 µl ddH_2_O. The PCR cycle consisted of an initial step of 94°C for 4 min, followed by 30 cycles of 94°C for 45 s, 55°C for 45 s, and 72°C for 1.0 min. An elongation step was carried out at 72°C for 10 min. A total of 30 cycles of PCR were performed. The PCR products (3 µl) were loaded on 1% agarose gel electrophoresis and confirmed through comparing the DNA sequence with NCBI database.

### 2.4 Salted duck egg white fermentation

The isolated strain C1 was activated and inoculated into the beef extract culture medium (3 g beef extract, 10 g peptone, and 50 g sodium chloride in 1,000 ml water with pH 7.4–7.6) for 24 hr for replication. Afterward, the strains were inoculated into salted duck egg white in a 5% weight‐to‐weight ratio and then fermented for 48 hr at 37°C in a fermentation incubator. After the fermentation, the resulted salted duck egg white sample was centrifuged at 4,000 rpm for 10 min to collect the sediments. The sediments were then immediately lyophilized and stored at 4°C before further analyses.

### 2.5 Determination of salt content in salted duck egg white before and after fermentation

The salted duck egg white sample after the fermentation was centrifuged at 4,000 rpm for 10 min to collect the precipitate. The resultant precipitate and the salted duck egg white sample were freeze‐dried. The freeze‐dried precipitate (5.0 g) was mixed with 100 ml distilled water in a glass flask with a stirrer. The mixture was well mixed under agitation and then kept in room temperature for 5 min. This process was repeated for 4 times. Afterward, 10 ml of the supernatant was transferred into a 50‐ml flask and then mixed with 20 ml distilled water. The mixture was then mixed with 8 drops of 5% K_2_CrO_4_ and then titrated using 0.2 M AgNO_3_ until the color mixture turned to be red. The content of sodium chloride in the egg white was calculated using the equation below,

NaCl%=(N×V×0.05845)/W×100%

where N and V represented the concentration (0.2 M) and volume of the AgNO_3_ solution used for the titration, whereas W indicated the weight of the sample (5.0 g). The molar weight of sodium chloride was 0.05845.

### 2.6 Differential scanning calorimetry

Differential scanning calorimetry of the control and fermented salted duck egg whites were analyzed using a 204F1 differential scanning calorimeter (Netzsch). Before experiment, the temperature and heat capacity of the calorimeter were calibrated using indium and sapphire. The control salted duck egg white and fermented salted duck egg white (10–15 mg) were respectively weighed onto aluminum differential scanning calorimetry pans. During the experiment, the heating rate of the differential scanning calorimeter scan was set at 1°C/min under a range of 30–100°C. The same aluminum pan with water was used as the reference. Each sample was measured in triplicate, and the differential scanning calorimetry data were analyzed using Universal Analysis Software for thermal analysis.

### 2.7 Scanning electron microscopy

An S‐3700N scanning electron microscope (Hitachi) was used to visualize the morphology and microstructure of the control and fermented salted duck egg whites. Both control and fermented salted duck egg white samples were lyophilized and then coated using a gold–palladium alloy coater and then observed on microscope under a 5,000‐time magnification with an accelerating voltage at 1.0 kV.

### 2.8 Amino acids determination

To determine the amino acids content, the control or fermented salted duck egg white sample (1 ml) was mixed with 10 ml 6 M HCl solution in an acid hydrolysis tube. The mixture was cooled in ice water for 5 min and then filled with nitrogen. Afterward, the resultant mixture was incubated at 110°C in an oven for 22 hr. After the incubation, the mixture was transferred to a 50‐ml volumetric flask and diluted to the volume using 0.02 M HCl solution. The extract was then filtered through 0.22 µm filter prior to amino acids analyzer.

For the analysis of free amino acids, the control or fermented salted duck egg white sample (1 ml) was mixed with 10 ml of 0.02 M HCl solution. The mixture was sonicated for 15 min and then deproteinized using an equal volume of 3% sulfosalicylic acid solution. Afterward, the mixture was transferred to a 50‐ml volumetric flask and diluted to the volume using 0.02 M HCl solution. The extract was then filtered through 0.22 µm filter prior to amino acids analyzer.

A Hitachi L‐8900 automatic amino acid analyzer connected with a Hitachi HPLC column and an ion‐exchange resin 2622 PF (60 × 4.6 mm, 3 µm, Hitachi, Japan) was used to analyze the amino acids content in these samples (Gong et al., 2017). The filtered sample (20 µl) was loaded on the analyzer based on the standard protocol of the manufacture. Amino acids present in these egg white samples were identified and quantified using their corresponding external standard. Each amino acid content was expressed as mg/g of sample weight.

### 2.9 γ‐Aminobutyric acid determination

The determination of γ‐aminobutyric acid followed a published method with minor modifications (Gong et al., 2017). The control or fermented salted duck egg white sample (100 µl) was mixed with 100 µl of 0.5M NaHCO_3_ solution and 200 µl of 4 g/L dansyl chloride‐acetone. The resultant mixture was incubated at 40°C for 1 hr and then filtered through 0.22 µm filters prior to HPLC analysis.

A Waters HPLC system was used to analyze γ‐aminobutyric acid in these samples (Waters Corporations) using a Hypersil BDS C18 column (250 mm × 4.6 mm, 5 µm). The injection volume was 10 µl, and the flow rate was set at 1.0 ml/min. The mobile phase consisted of (A) methanol and (B) 5:75: 420 tetrahydrofuran: methanol: 0.05M CH_3_COONa (v/v/v). The column was maintained at 25°C during gradient program, and the gradient was as follows: 0–5 min, 80%B isocratic; 5–20 min, 80%B to 50%B; 20–21 min, 50%B to 0%B; 21–27 min, 0%B isocratic; 27–28 min, 0%B to 80%B; and 28–30 min, 80%B isocratic. The wavelength of visible light detector was set at 254. The external γ‐aminobutyric acid was used for quantification and expressed as mg/g of sample weight.

### 2.10 Statistical analysis

Data were expressed as the mean ± *SD* of triplicate tests. One‐way analysis of variance under Duncan's multiple range test was used to determine the significant differences among the mean at a significant level of 0.05 under SPSS 11.0 statistical software.

## 3 RESULTS AND DISCUSSION

### 3.1 Strain Isolation and Screening

Three haloduric bacterial strains were isolated from Jinhua ham using International Streptomyces Project (ISP) 2 medium complemented with 10% sodium chloride. Furthermore, these strains were cultured in the ISP medium 3 and 4 to investigate their protease‐producing activity and the ratio of the transparent circle diameter to the colony diameter (HC) and gelatin liquefaction capacity, respectively. Table 1 exhibits their protease‐producing activity and HC and gelatin liquefaction capacity. It was found that these strains displayed a 1.1–1.3 mm colony diameter with their transparent circle ranging of 2.5–2.7 mm. It should be worth noting that the strain C2 and C3 showed their HC value of 1.92 and 2.08, respectively. However, the strain C1 possessed a much higher value on the HC compared with the other strains. Meanwhile, the strain C1 also exhibited the highest capacity on the gelatin liquefaction compared with the strain C2 and C3. These indicated that the strain C1 was screened as the best strain that could ferment salted duck egg white compared to the other isolated strains.

**Table 1:** Colony diameter, transparent circle, HC, and gelatin liquefaction of three bacterium strains isolated from Jinhua ham

Parameters	Strain		
	C1	C2	C3
Colony diameter (mm)	1.1 ± 0.10	1.3 ± 0.17	1.2 ± 0.17
Transparent circle (mm)	2.7 ± 0.17	2.5 ± 0.26	2.5 ± 0.10
Transparent Circle to Colony Diameter Ratio (HC)	2.45 ± 0.12	1.92 ± 0.07	2.08 ± 0.06
Gelatin liquefaction	+++	++	++

Note

Data are mean ± *SD* (*n* = 3). +++ and ++ represent positive and weakly positive, respectively.

### 3.2 Strain identification and characterization

Since the strain C1 appeared to be the best bacteria regarding its HC and gelatin liquefaction capacity. The physiological and biochemical feature of the strain C1 was further investigated. Regarding the feature of the C1 thallus and liquid culture, the growth of the strain C1 resulted in a significant turbidity and the strain C1 was precipitated at the bottom of the medium with the mannitol salt agar broth in the yellow color. Furthermore, the strain C1 individual cell exhibited a spherical form with a crumbly structure and a grape‐bunch‐like shape. The appearance of the strain C1 colony appeared to be white and round, and its texture was moist smooth with an up to 1 mm colony diameter. The strain C1 was found to be a gram‐negative bacterium with a pH tolerance till 9. Meanwhile, it could be salt resistant with the salt level up to 20% sodium chloride (w/v). This strain was confirmed to prefer glucose and saccharose as its carbon resources with an ability of consuming mannitol and xylose. Tryptone and beef extract were their primary nitrogen source. Moreover, the strain C1 also possessed the catalase and had capacities of glucose oxidation, gelatin liquefaction, sucrose oxidation, starch oxidation, and nitrate reduction (Table 2).

**Table 2:** Physiological and biochemical features of haloduric bacterium strain C1

Parameters	Strain C1
Gram stain	G^+^
pH tolerence	
9	+
10	−
11	−
Temperature	
45°C	−
NaCl (%, w/v)	
10	+
15	w
20	w
25	−
Use of sole carbon sources (1%, w/v)	
Glucose	+
Saccharose	+
Mannitol	w
Xylose	w
Citric acid	−
Sodium carbonate	−
Use of sole nitrogen sources (0.1%, w/v)	
Tryptone	+
Beef extract	+
Yeast powder	w
Potassium nitrate	−
Ammonium sulfate	−
Ammonium chloride	−
Biochemical feature	
Catalase	+
Glucose oxidation	+
Liquefaction of gelation	+
Methyl red	−
Sucrose oxidation	+
Starch oxidation	+
Nitrate reduction	+

Note

“+” indicates positive, whereas “−” represents negative. “w” indicates weakly positive.

**Figure 1:**
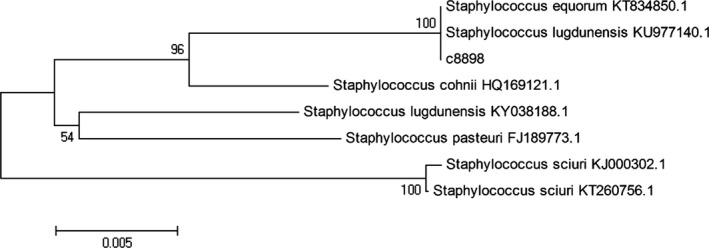
Neighbor‐joining tree based on almost complete 16S rRNA gene sequences showing the position of C1 among its phylogenetic neighbors Asterisks indicate branches of the tree also recovered using maximum‐likelihood and maximum‐parsimony tree‐making algorithms. Numbers at nodes indicate levels of bootstrap support (%), with only values ≥50% shown

In order to identify the strain C1, we further investigated the 16S rRNA gene sequence of the strain C1 using the EzTaxon‐e analysis (Figure 1). It was found that the strain C1 belonged to the genus *Staphylococcus* due to the similarity with some members of *Staphylococcus* using the neighbor‐joining algorithm under the phylogenetic tree. Particularly, this strain shared more similarity with *Staphylococcus equorum* KT834850.1 and *Staphylococcus lugdunensis* KU977140.1, indicating that the strain C1 possessed the close relation with those strains (Priest, Goodfellow, & Todd, 1988).

### 3.3 Salt alteration in salted duck egg white after fermentation

The salt content of the egg white precipitate significantly decreased before and after the fermentation. It was found that the salt content in the salted duck egg white (before the fermentation) was about 38.5%. After the fermentation, the egg white precipitate contained about 15.0% salt content, indicating that the fermentation significantly lowered the salt content in the egg white. It has been reported that proteinases released from the bacteria could damage the structure of protein, which could further result in the collapse of the colloidal network formed between protein and water molecules (Signorini, Salazar, Ponce‐alquicira, & Guerrero‐legarreta, 2010). As a result, water released from the colloidal network could interact with salt to lower the concentration of salt in egg white protein. With the fermentation, protein molecules could further be degraded into peptides and the alteration of pH condition could accelerate the phase separation in the egg white suspension.

### 3.4 Differential scanning calorimetry (DSC) of salted duck egg white

**Figure 2:**
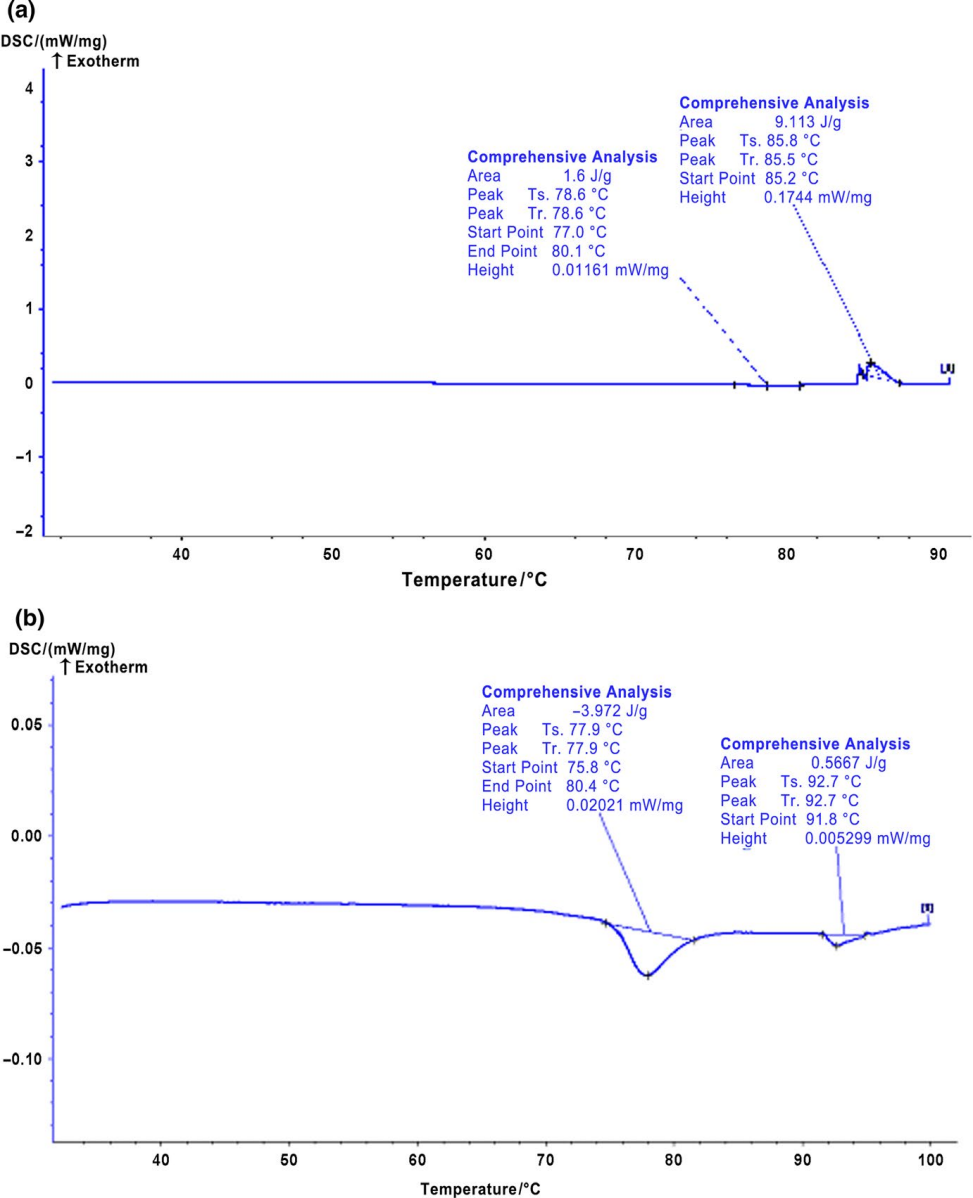
Differential scanning calorimetry of (a) control and (b) fermented salted duck egg white

Differential scanning calorimetry (DSC) is a common technique to evaluate the thermodynamic information on protein denaturation through measuring the endothermic and exothermic changes of protein (Briere, Brandt, & Medley, 2010). Figure 2 shows the DSC thermograms of the control and the strain C1 fermented salted duck egg white samples, whereas the denaturation temperatures of these egg white samples are summarized in Table 3. It was found that the control sample (salted duck egg white without fermentation) exhibited two major endothermic peaks with one at 77–79°C and the other at 85–93°C (Figure 2). It has been reported that ovomucoid and ovalbumin were denatured at 77°C and 84°C, respectively (Donovan, Mapes, Davis, & Garibaldi, 1975). Therefore, we speculated that those two major endothermic peaks in the control salted duck egg white resulted from the denaturation of ovomucoid and ovalbumin since these two proteins are the major protein components in duck egg white. Compared with the control salted duck egg white, the strain C1 fermented salted duck egg white sample had a similar endothermic peaks temperature at around 77°C. This was caused by the denaturation of ovomucoid in the fermented salted duck egg white. This indicated that the strain C1 did not induce any structural alteration on ovomucoid in the egg white. It should be worth noting that fermentation of the salted duck egg white with the strain C1 resulted in a disappearance of the endothermic peaks at around 85°C (Table 3 and Figure 2). This demonstrated that the denaturation of ovalbumin in the egg white altered after the fermentation, which indicated that the fermentation process resulted in structural alteration and/or disintegration of ovalbumin in the egg white (Ma & Harwalkar, 1991). It was accepted that the enthalpy of protein denaturation was caused mainly by the alteration of protein secondary and tertiary structures. The main force in protein secondary structure included hydrogen bonds, hydrophobic interaction, and ionic strength, whereas the tertiary structure of protein was mainly aggregation of proteins/peptides. Therefore, we speculated that the fermentation might disrupt these interactions in ovalbumin in the egg white, resulting in its disappearance of its endotherm peak. Surprisingly, a new endotherm peak was found in the strain C1 fermented salted duck egg white sample starting at 91.8°C. It was suggested that the fermentation might induce the aggregation and/or rearrangement of the proteins/peptides in egg white, which provided the fermented salted duck egg white with a new endotherm feature (Thomas & Waldemar, 2010).

**Table 3:** Calorimetric measurement of control and fermented salted duck egg white denaturation

Sample	*T*_01_ (°C)	*T*_P1_ (°C)	*T*_C1_ (°C)	Δ*H* (J/g)	*T*_02_ (°C)	*T*_P2_ (°C)	*T*_C2_ (°C)	Δ*H* (J/g)
Control	77.0	78.6	80.4	−1.60	85.2	85.8	88.0	9.1
Fermented	75.8	77.9	80.4	−3.97	91.8	92.7	95.8	−0.6

### 3.5 Scanning electron microscopy (SEM) of salted duck egg white

**Figure 3:**
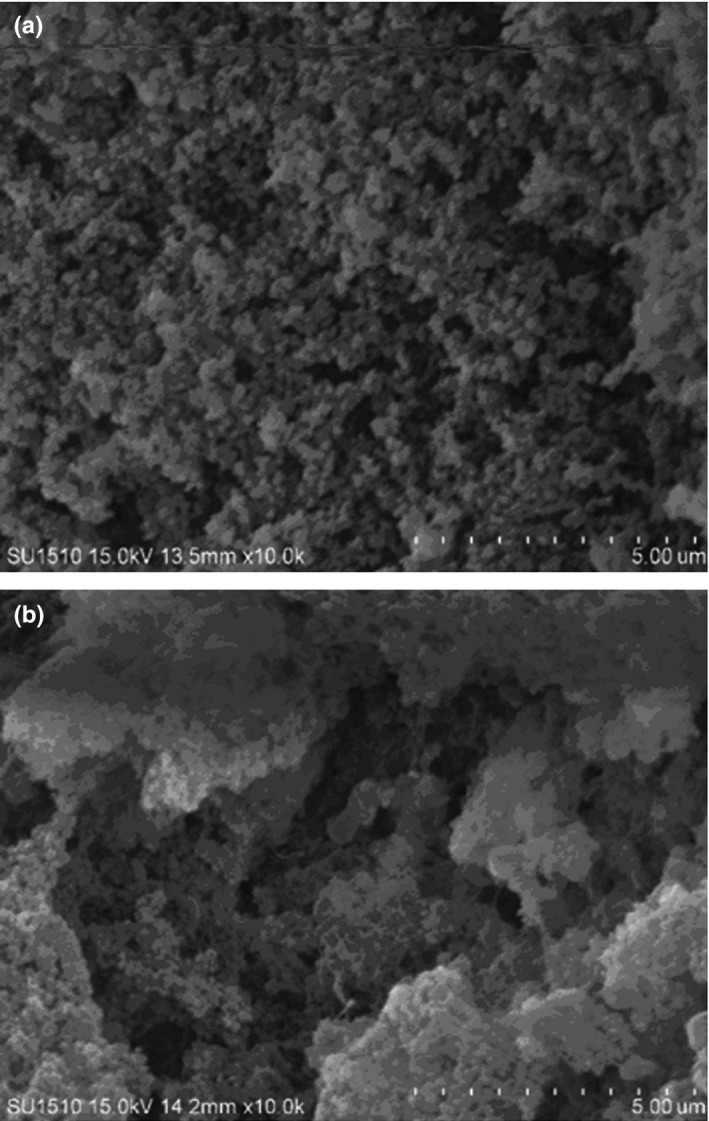
Scanning electron microscopy of (a) control and (b) fermented salted duck egg white

Scanning electron microscopy is normally used to visualize the morphological feature of protein, which could help indicate the overall quality of protein and protein products (Gorinstein et al., 2010). Figure 3 exhibits the morphology of the control and the strain C1 fermented salted duck egg white samples. The salted duck egg white sample exhibited the uniform pores on the surface. However, the strain C1 fermentation resulted in the salted duck egg white with harder and denser in the gel microstructure. Our results were consistent with the previous study (Zheng, Zeng, Kan, & Zhang, 2018). It should be also noted that the fermentation also decreased the integrity of the salted duck egg white structure, which might result from the irreversible disruption and rearrangement of protein molecules in the egg white.

### 3.6 Amino acids composition and γ‐aminobutyric acid

It is known that microorganisms during fermentation process could release various enzymes that could hydrolyze proteins and peptides into amino acids (Gong et al., 2017, 2019). In the present study, the control salted duck egg white was not found to contain free amino acids. The total amino acids content in the control egg white was about 549 mg/g (Table 4). Glutamic acid, aspartic acid, leucine, serine, phenylalanine, valine, methionine, and lysine appeared to be the major amino acids in the control salted duck egg white sample. The salted duck egg white after the fermentation by the strain C1 contained much higher concentration on the total amino acids (852 mg/g). This was because the autolysis of these strains could release more amino acids in the salted duck egg white (Crow et al., 1995; Hickey, Ross, & Hill, 2004; Saleh, Zhang, & Shen, 2016). Meanwhile, glycine, aspartic acid, glutamic acid, alanine, and proline were found to be the dominant amino acids in the fermented egg white sample, and their content in the fermented egg white was much higher than that in the control. Additionally, various enzymes released from the strain C1 might play an important role in hydrolyzing the protein components in the salted duck egg white during fermentation, which might result in the release of some free amino acids. It was found that the salted duck egg white fermented by the strain C1 contained the total free amino acids content of about 38 mg/g. Meanwhile, the free form serine appeared to be the dominant free amino acid in the egg white, followed by glycine, alanine, arginine, and aspartic acid. It should be worth noting that the control salted duck egg white did not contain γ‐aminobutyric acid. However, the fermented salted duck egg white was found to contain γ‐aminobutyric acid. It has been reported that γ‐aminobutyric acid could be primarily formed from glutamate via glutamate decarboxylase and is a major inhibitory neurotransmitter for the central nervous system. This compound could reduce the risk of depression and improve immune system of human body (Gong et al., 2017, 2019). Therefore, the nutritional quality of the salted duck egg white might be significantly enhanced by the fermentation of the strain C1.

**Table 4:** Amino acids content and γ‐aminobutyric acid content in control and fermented salted duck egg white

Salted duck egg white (mg/g)	Amino acids composition		Free form amino acids composition	
	Control	Fermented	Control	Fermented
Aspartic acid	53.19 ± 2.01	131.05 ± 1.79	ND	3.21 ± 0.07
Threonine	31.34 ± 0.66	25.40 ± 1.11	ND	1.44 ± 0.05
Serine	43.85 ± 0.59	56.60 ± 0.90	ND	8.79 ± 0.22
Glutamic acid	87.72 ± 095	117.21 ± 0.80	ND	2.16 ± 0.07
Glycine	21.27 ± 1.96	164.23 ± 0.74	ND	7.10 ± 0.12
Alanine	25.75 ± 2.48	55.54 ± 0.39	ND	5.04 ± 0.04
Cysteine	3.32 ± 0.20	3.74 ± 0.31	ND	ND
Valine	38.13 ± 2.39	26.13 ± 0.92	ND	2.17 ± 0.06
Methionine	37.10 ± 0.70	35.13 ± 0.65	ND	ND
Isoleucine	22.52 ± 1.08	13.15 ± 0.99	ND	1.19 ± 0.04
Leucine	50.29 ± 2.04	34.81 ± 0.58	ND	1.91 ± 0.04
Tyrosine	16.43 ± 1.22	28.65 ± 0.38	ND	ND
Phenylalanine	37.10 ± 1.22	25.86 ± 0.71	ND	ND
Lysine	30.19 ± 0.54	31.43 ± 0.60	ND	1.37 ± 0.03
Histidine	10.19 ± 0.79	18.78 ± 0.82	ND	ND
Arginine	27.17 ± 1.12	35.56 ± 0.79	ND	4.09 ± 0.07
Proline	13.55 ± 0.63	49.06 ± 0.29	ND	ND
γ‐aminobutyric acid	ND	4.26 ± 0.23	ND	3.94 ± 0.08

Note

Data are mean ± *SD* (*n* = 3). “ND” represent “not detected”.

## 4 CONCLUSION

In conclusion, three haloduric bacteria strains were isolated from Jinhua ham. The haloduric bacterium strain C1 appeared to be the best strain due to its high HC and gelatin liquefaction. The salted duck egg white fermented by the strain C1 reduced its salinity. Meanwhile, the fermented egg white increased its thermal stability as confirmed by differential scanning calorimetry analysis compared to the control salted duck egg white without fermentation. Scanning electron microscopy revealed that the microstructure of the salted duck egg white fermented by the strain C1 was harder and denser than the control, and its integrity was reduced after the fermentation. Free amino acids and γ‐aminobutyric acid were detected in the salted duck egg white after the fermentation by the strain C1, whereas the control did not contain these nutrients.

## CONFLICT OF INTEREST

All authors declared that they have no conflict of interest.

## ETHICAL APPROVAL

This study does not involve any human or animal testing.

## INFORMED CONSENT

Written informed consent was obtained from all study participants.
